# Characterization of the complete chloroplast genome sequence of the giant knotweed, *Fallopia sachalinensis* from the volcanic island Dokdo, Republic of Korea

**DOI:** 10.1080/23802359.2019.1663769

**Published:** 2019-09-12

**Authors:** Gurusamy Raman, Kye Tae Park, Gi Heum Nam, Myounghai Kwak, SeonJoo Park

**Affiliations:** a Department of Life Sciences, Yeungnam University, Gyeongsan, Republic of Korea;; b Plant Resources Division, National Institute of Biological Resources of Korea, Incheon, Republic of Korea

**Keywords:** *Fallopia sachalinensis*, chloroplast genome, next-generation sequencing, Polygonaceae, giant knotweed

## Abstract

The giant knotweed plant, *Fallopia sachalinensis* is confined to Ulleung and Dokdo islands, Korea. Here, we reported the complete chloroplast genome of* F. sachalinensis*. The chloroplast genome size was 163,485 bp in length, containing a couple of identical inverted repeat regions of 31,108 bp, a large single-copy region of 87,703 bp and small single-copy region of 13,566 bp. The genome encoded 129 genes, of which 112 were unique, including 78 protein-coding, 30 tRNA and 4 rRNA genes. The maximum-likelihood phylogenetic tree showed that F. sachalinensis is a basal group and sister to the rest of the Polygonaceae family plants.

*Fallopia sachalinensis* (F. Schmidt) Ronse Decr. is also called as giant knotweed belongs to sect. *Reynoutria* (Houtt.) Ronse Decr. in the Polygonaceae family. Due to the presence of erect robust stems, well-developed thick rhizomes, deeply three-parted styles with the fimbriate stigma, large orbicular to broadly ovate leaves acuminate to cuspidate apices and dioecious breeding system, this species is distinct from other section in the genus (Decraene and Akeroyd 1988; Kim and Park 2000; Park et al. 2018). Section *Reynoutria* includes 12 species that are naturally distributed in Asia such as China, Japan, Korea, and Russia (Kim and Park 2000). Among the section, three species namely *F*. *sachalinensis*, *F. japonica* and *F. forbesii* (Hance) Yonekura & H. Ohashi are living in Korea (Kim and Park 2000; Park et al. 2018). The species, *F. sachalinensis* occurs naturally in East Asia specifically in China, Korea, northern and central areas of Japan, Sakhalin and Kuril island of Russia (Balogh 2008). In Korea, this species is restricted to Ulleung and Dokdo islands (Park et al. 2018). Interestingly, the species widely distributed in Ulleung Island, whereas a single large population is found in the volcanic island, Dokdo. Though previous studies were carried out to understand the colonization events and phylogenetic relationships with few chloroplast genes, there is no inclusive study about the complete chloroplast genome sequence of *F. sachalinensis* (Park et al. 2018). So, in the present study, we collected *F. sachalinensis* plant from Dokdo Island (geospatial coordinates: N37°14′30″, E131°51′47″) and the specimen stored at National Institute of Biological Resources (NIBR), Republic of Korea (Specimen accession number: NIBRGR0000600282) and analyzed and characterized the complete chloroplast genome sequence.

The size of the complete cp genome of *F. sachalinensis* is 163,485 bp with GC content is 37.5% and shows a typical quadripartite structure, consisting of the large single-copy (LSC; 87,703 bp) and small single-copy (SSC; 13,566 bp) separated by a pair of inverted repeats (IRs; 31,108 bp) (GenBank: MK842154). The cp genome has encoded a total of 129 functional genes, of which 112 were unique and 17 duplicated in IR regions. The cp genome contains 78 unique protein-coding genes, 6 of which were duplicated in the IR region. Additionally, 30 unique tRNA genes were distributed throughout the genome.

In order to determine the phylogenetic relationships, in addition to *F. sachalinensis*, we included 13 species from Caryophyllales and *Nicotiana tabacum* species as the outgroup. The maximum-likelihood tree was constructed using the concatenated 76 cp protein-coding genes. The phylogenetic tree was comprised of two major clades such as all Polygonaceae species formed one clade and other species of Caryophyllales formed another clade (Figure 1). Within the Polygonaceae clade, *F. sachalinensis* is a basal group and sister to the rest of the species of Polygonaceae with maximum bootstrap value (100%).

**Figure 1.**
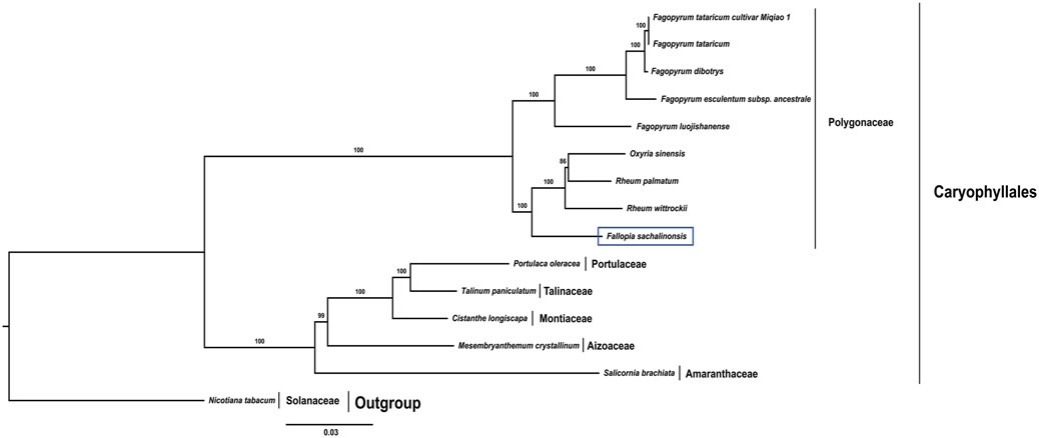
Molecular phylogenetic tree of 14 Caryophyllales taxa based on 76 protein-coding genes in the chloroplast genome. The tree was constructed by maximum-likelihood analysis of the conserved regions using the RAxML program and the GTR + G + I nucleotide model. The stability of each tree node was tested by bootstrap analysis with 1000 replicates. Bootstrap values are indicated on the branches, and the branch length reflects the estimated number of substitutions per 1000 sites. *Nicotiana tabacum* was set as the outgroup.

In conclusion, the complete cp genome of *F. sachalinensis* may contribute to a better understanding of the phylogenetic relationships and also it may be useful in assessing the genetic diversity and molecular identification of the Polygonaceae family.
